# PID1 increases chemotherapy-induced apoptosis in medulloblastoma and glioblastoma cells in a manner that involves NFκB

**DOI:** 10.1038/s41598-017-00947-6

**Published:** 2017-04-11

**Authors:** Jingying Xu, Xiuhai Ren, Anup Singh Pathania, G. Esteban Fernandez, Anthony Tran, Yifu Zhang, Rex A. Moats, Gregory M. Shackleford, Anat Erdreich-Epstein

**Affiliations:** 1 Saban Research Institute at Children’s Hospital Los Angeles, Division of Hematology, Oncology and Blood & Marrow Transplantation, Department of Pediatrics, Los Angeles, California 90027 USA; 2 Saban Research Institute at Children’s Hospital Los Angeles, Cellular Imaging Core, Los Angeles, California 90027 USA; 3 Saban Research Institute at Children’s Hospital Los Angeles, Department of Radiology, Los Angeles, California 90027 USA; 4 Keck School of Medicine, University of Southern California, Departments of Pediatrics and Pathology, Los Angeles, California 90033 USA

## Abstract

Phosphotyrosine Interaction Domain containing 1 (PID1; NYGGF4) inhibits growth of medulloblastoma, glioblastoma and atypical teratoid rhabdoid tumor cell lines. PID1 tumor mRNA levels are highly correlated with longer survival in medulloblastoma and glioma patients, suggesting their tumors may have been more sensitive to therapy. We hypothesized that PID1 sensitizes brain tumors to therapy. We found that PID1 increased the apoptosis induced by cisplatin and etoposide in medulloblastoma and glioblastoma cell lines. PID1 siRNA diminished cisplatin-induced apoptosis, suggesting that PID1 is required for cisplatin-induced apoptosis. Etoposide and cisplatin increased NFκB promoter reporter activity and etoposide induced nuclear translocation of NFκB. Etoposide also increased PID1 promoter reporter activity, PID1 mRNA, and PID1 protein, which were diminished by NFκB inhibitors JSH-23 and Bay117082. However, while cisplatin increased PID1 mRNA, it decreased PID1 protein. This decrease in PID1 protein was mitigated by the proteasome inhibitor, bortezomib, suggesting that cisplatin induced proteasome dependent degradation of PID1. These data demonstrate for the first time that etoposide- and cisplatin-induced apoptosis in medulloblastoma and glioblastoma cell lines is mediated in part by PID1, involves NFκB, and may be regulated by proteasomal degradation. This suggests that PID1 may contribute to responsiveness to chemotherapy.

## Introduction

Phosphotyrosine interaction domain containing 1 (PID1, NYGGF4, Gene ID: 55022) was identified in 2006^1^ and found to be an inhibitor of insulin-mediated signaling in adipocytes and muscle cells^2–4^. PID1 has been linked to obesity, insulin resistance, Alzheimer’s disease and cancer^1, 5–7^. PID1 is mostly known for its inhibition of insulin receptor signaling, impairment of mitochondrial function and binding through its phosphotyrosine binding (PTB) domain to the second NPXY motif in the cytoplasmic tail of the low density lipoprotein receptor-related protein 1 (LRP1)^1–5, 8–12^.

We recently linked PID1 to brain tumors and to cancer by establishing that PID1 is growth inhibitory in medulloblastoma, glioma and atypical teratoid rhabdoid tumor (ATRT) cell lines^7^. PID1 decreased proliferation, induced apoptosis, impaired mitochondrial membrane potential, and inhibited phosphorylation of AKT and ERK in medulloblastoma and glioblastoma (GBM) cell lines^7^. Clinically, levels of PID1 mRNA in medulloblastoma and glioma tumors directly correlate with patient survival^7^. Consistent with this, the more aggressive subgroups of medulloblastomas (molecular subgroups 3 and 4) and gliomas (GBMs, especially mesenchymal GBM) have lower PID1 mRNA compared to more favorable medulloblastomas and gliomas^7^. Importantly, PID1 contributes to a new 31-gene medulloblastoma molecular subgroup classification score, further supporting its relevance in medulloblastoma biology^13, 14^.

The longer survival of patients whose medulloblastoma and glioma tumors at diagnosis contained higher *PID1* mRNA^7^ suggested that these patients’ tumors may be more responsive to therapy and raised the possibility that PID1 may sensitize medulloblastomas and gliomas to chemotherapy. Our work here shows that PID1 augments the apoptotic effect of etoposide and cisplatin in medulloblastoma and glioma cell lines and that PID1 is required for part of the cisplatin-induced apoptosis of medulloblastoma cells. The mechanism involves chemotherapy-induced, NFκB-dependent increase in PID1 mRNA. Interestingly, while the etoposide-induced increase in *PID1* mRNA was associated with increased PID1 protein, the cisplatin-induced *PID1* mRNA increase was associated with a decrease in PID1 protein that was reversed by the proteasomal degradation inhibitor bortezomib. Thus, PID1 sensitizes medulloblastomas and gliomas to etoposide and cisplatin, and mediates at least part of the chemotherapy response.

## Results

We previously showed that higher tumor *PID1* mRNA correlates with longer overall survival in glioma and medulloblastoma patients^7^. An additional independent glioma dataset (Supplementary Fig. S1) and an expanded GBM patient cohort (TCGA, n = 504; Supplementary Fig. S1) show similar correlations, further supporting our prior findings. We therefore hypothesized that PID1 may sensitize gliomas and medulloblastoma cells to therapy.

### Overexpression of PID1 sensitizes brain tumor cells to cisplatin and etoposide

**Figure 1:**
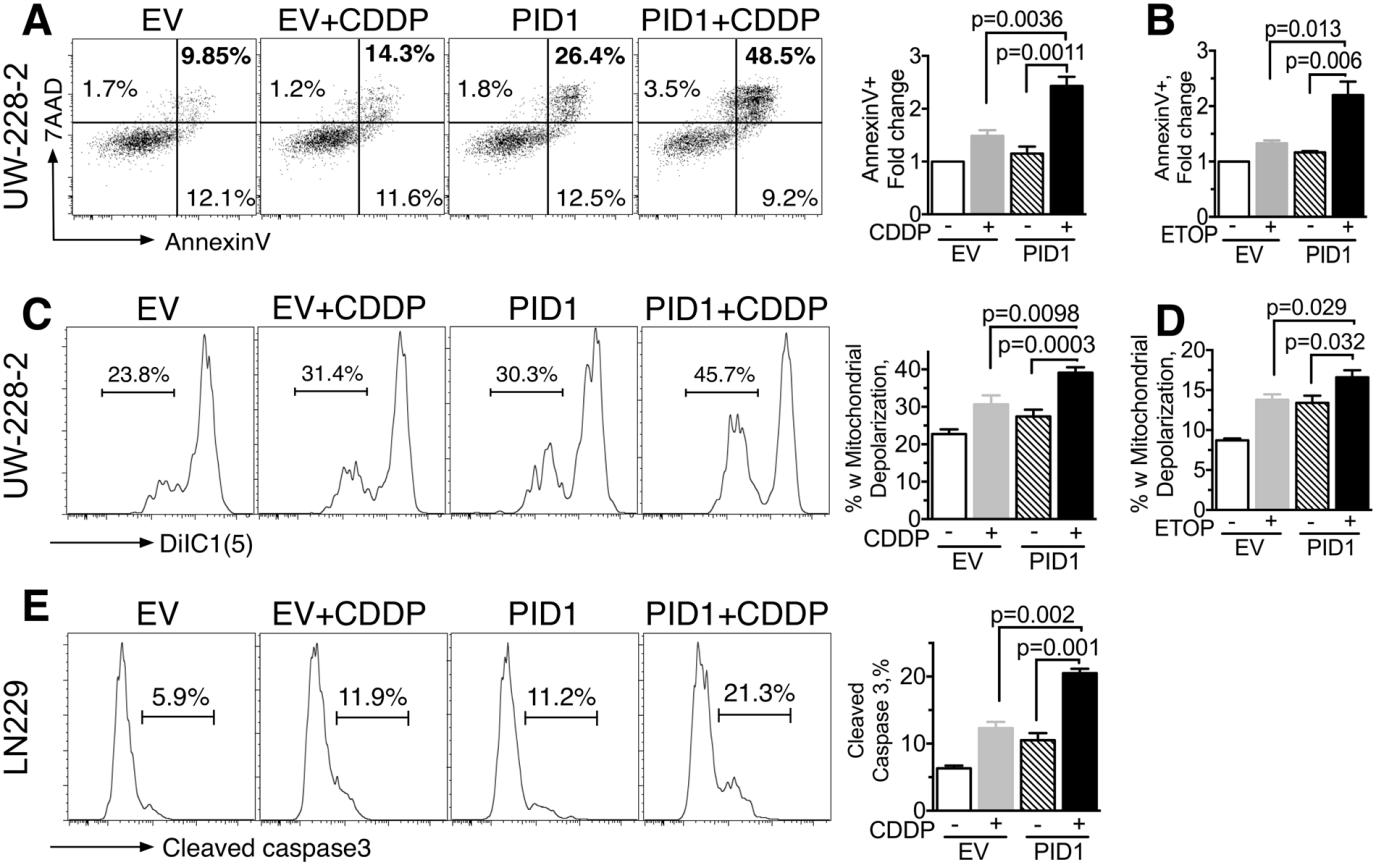
PID1 enhances apoptosis of brain tumor cells treated with cisplatin or etoposide. Medulloblastoma (UW228) and GBM (LN229) cells (4 × 10^5^ per well) were seeded in 6-well plates overnight and were transfected with 1 µg eGFP-containing pCIENS-PID1 or empty vector control for 6 hrs. The next day cells were treated with cisplatin (CDDP, 10 µg/ml; (**A**,**C**,**E**) or etoposide (ETOP, 5 µg/ml, (**B**,**D**,**F**) in 2 ml complete medium for 24 hrs. Apoptosis in the GFP-positive (transfected) cells was assessed by flow cytometry. (**A**,**B)** AnnexinV 7AAD in UW228 cells; values shown are for the upper and lower right quadrants, indicative of early + late apoptosis (7AAD+/AnnexinV- quadrant indicative of necrosis was negligible); (**C**,**D)** DiIC1(5), indicative of mitochondrial depolarization, in UW228 cells. (**E)** Cleaved caspase-3 in LN229 cells. For panels (A,C,E), data from a single experiment is shown on left and mean ± SEM summary data from 2–5 individual experiments, most performed in 2–4 replicates, are shown in the bar graphs on the right. Western blot of PID1 protein expression from one of the experiments contributing to panels A–D is shown in Supplementary Fig. S6B. Similar findings in D283 medulloblastoma and LN229 GBM cells are shown in Supplementary Fig. S2.

We first compared apoptosis induced by cisplatin or etoposide in cells that transiently overexpressed either vector control or PID1 (Fig. 1). UW228 medulloblastoma cells simultaenously expressing PID1 and treated with cisplatin showed higher AnnexinV staining and greater mitochondrial membrane depolarization compared to control or single treatment cells (Fig. 1A and C). Similar results were seen in D283 medulloblastoma cells and LN229 GBM cells (Supplementary Fig. S2). A second chemotherapy drug, etoposide, when similarly combined with PID1 in UW228 cells also showed increases in AnnexinV and mitochondrial membrane depolarization, which were larger than for either etoposide or PID1 alone (Fig. 1B and D). Similar results were seen in LN229 GBM cells transfected with PID1 and treated with etoposide (Supplementary Fig. S2). Caspase-3 cleavage, another measure of apoptosis, was also higher in LN229 GBM cells transfected with PID1 and treated with cisplatin compared to vehicle and empty vector controls (Fig. 1E). Thus PID1 augmented the apoptotic effect of cisplatin and etoposide in medulloblastoma and GBM cell lines, suggesting sensitization of brain tumor cell lines to chemotherapy by PID1.

### siRNA to *PID1* mitigates apoptosis and mitochondrial depolarization induced by cisplatin

**Figure 2:**
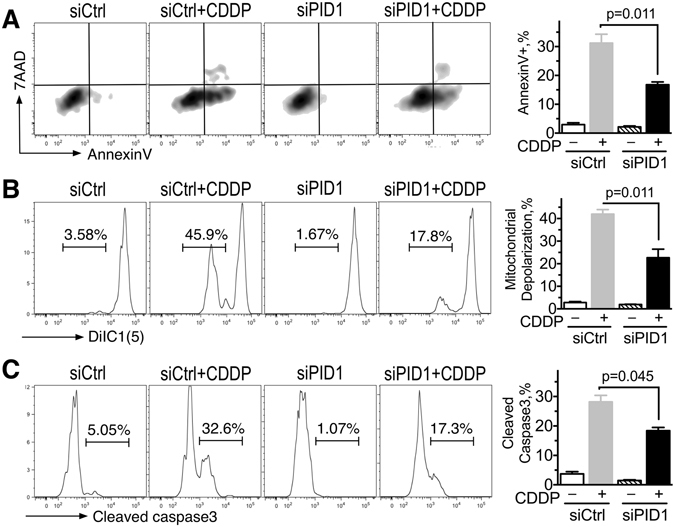
Knockdown of PID1 diminishes apoptosis induced by cisplatin. UW228 cells (4 × 10^5^ per well) were transfected with non-silencing control siRNA (siCtrl) or PID1 siRNA (siPID1) and FAM-labeled non-silencing siRNA to label transfected cells. Twenty-four hrs after transfection cells were treated with 10 µg/ml cisplatin for 24 hrs, and apoptosis (AnnexinV positive (**A**), mitochondrial membrane depolarization (DiIC1(5)) (**B**), or cleaved caspase-3 (**C**) were assessed by flow cytometry in the FAM-labeled cells. A representative flow cytometry curve is on the left and mean ± SEM (n = 3 biological replicates) of one experiment of two with similar results are shown on the right. Western blot of PID1 siRNA knockdown is shown in Supplementary Fig. S3D. Similar effects on mitochondrial membrane depolarization were seen in D283 medulloblastoma and U87 GBM cells (see Supplementary Fig. S3).

Supporting the increased apoptosis in presence of PID1 + chemotherapy, siRNA-mediated PID1 knockdown had the opposite effect and diminished the cisplatin-induced apoptosis (AnnexinV binding, caspase-3 cleavage) and depolarization of mitochondrial membrane potential in UW228 medulloblastoma cells compared to cells treated with cisplatin + non-silencing control siRNA (Fig. 2; for PID1 protein level after si*PID1* knockdown see Supplementary Fig. S3D). D283 medulloblastomas and U87 glioma cells treated with si*PID1* showed similar decrease in the cisplatin effect on depolarization of mitochondrial membrane potential (Supplementary Fig. S3). This suggests that cisplatin-induced apoptosis and mitochondrial depolarization in medulloblastoma and glioma cells occur, at least partially, via a mechanism that requires PID1.

### Chemotherapy increases *PID1* mRNA

**Figure 3:**
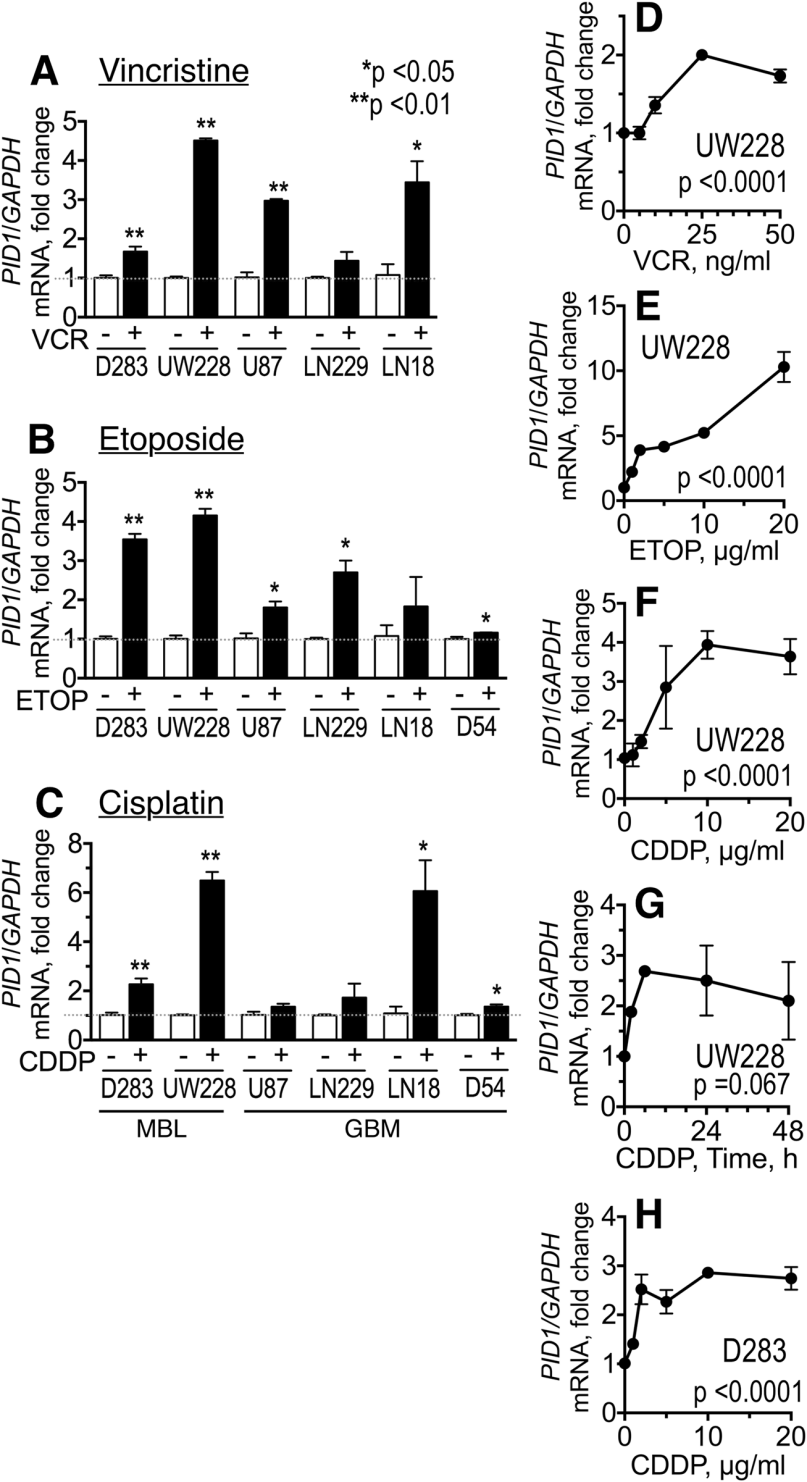
Chemotherapy can increase PID1 mRNA. (**A**–**C)** Medulloblastoma (D283MED, UW228) and glioblastoma (U87, LN229, LN18, D54) cell lines (4 × 10^5^ cells/6-well) were treated for 24 hrs with vincristine (**A**: 50 ng/ml), etoposide (**B**: 5 µg/ml in D283, UW228, U87, LN229, LN18; 10 µg/ml in D54), or cisplatin (**C**: 5 µg/ml in D283, U87, LN229, LN18; 10 µg/ml in UW228-2 and D54). (**D**–**F)** UW228 cells were treated with increasing doses of vincristine (VCR, panel D), etoposide (ETOP, panel E) or cisplatin (CDDP, panel F) for 24 hrs. (**G)** UW228 cells were treated with 5 µg/ml cisplatin for the times indicated. (**H)** D283 medulloblastoma treated as in (**F**). In all panels, cells were collected at end of incubation, RNA extracted, and qRT-PCR for *PID1* mRNA analyzed. Experiments were performed in triplicate up to three times. Shown are fold change in *PID1* mRNA relative to *GAPDH* mRNA of chemotherapy-treated compared to vehicle-treated cells, normalized to 1.

*PID1* mRNA level in 3T3-L1 adipocytes is increased by TNFα and free fatty acids and decreased by IL-6 and leptin suggesting that stressors could affect PID1 expression^15^. We examined effect of cisplatin, etoposide, and vincristine on *PID1* mRNA level in UW228 and D283MED medulloblastoma cell lines and LN229, U87, LN18, and D54 GBM cell lines. The three drugs increased *PID1* mRNA in these cell lines to varying degrees with the increase in *PID1* mRNA being dose-dependent and time-dependent (Fig. 3). Peak *PID1* mRNA level was reached after as little as 8 hrs of cisplatin (5 µg/ml) and was still high at the completion of the experiment at 48 hrs. Thus, chemotherapy can increase *PID1* mRNA in brain tumor cell lines.

### Chemotherapy-induced increase in *PID1* mRNA is associated with nuclear translocation of NFκB and is blocked by NFκB inhibitors

**Figure 4:**
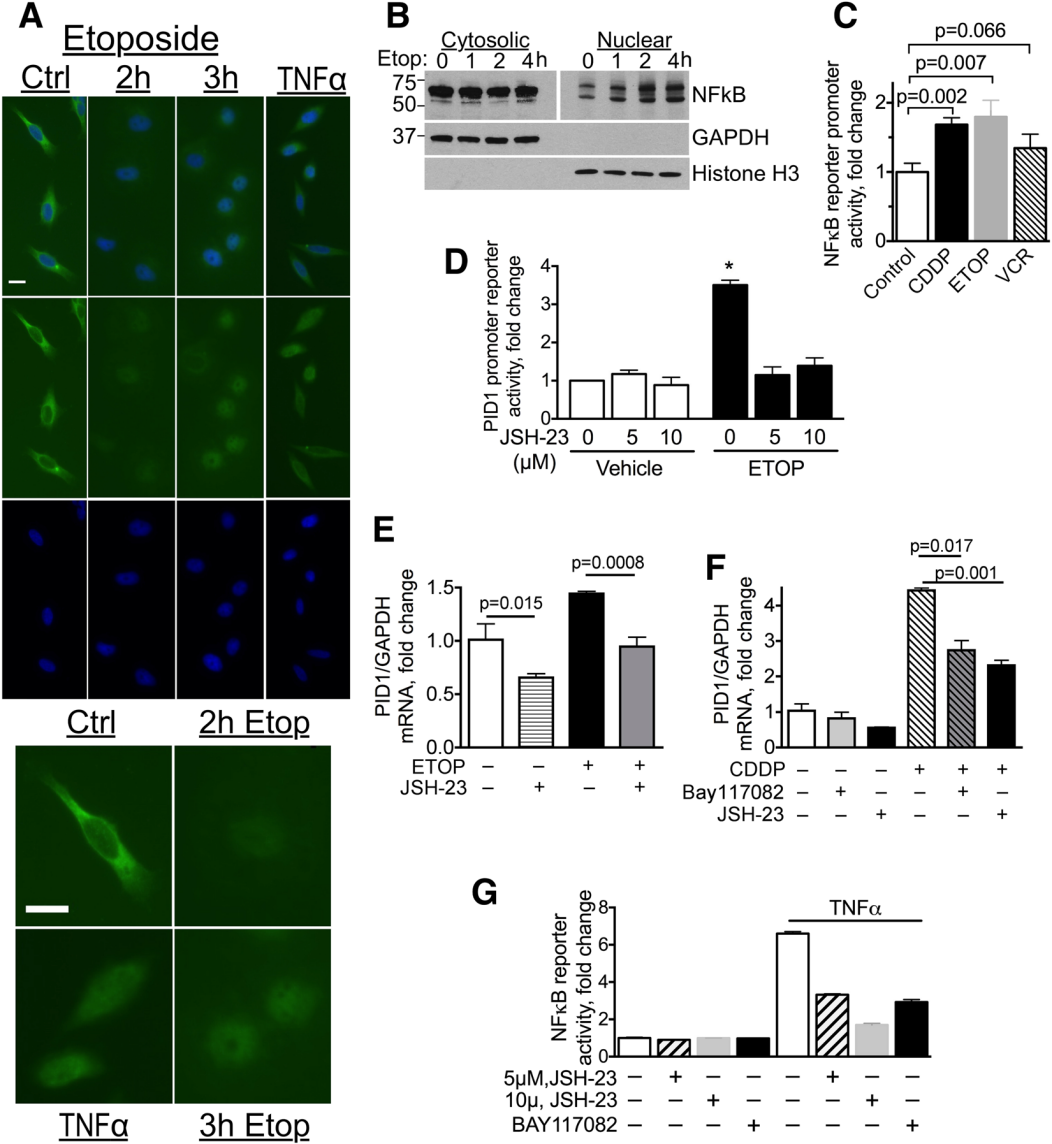
Chemotherapy induces nuclear translocation of NFκB and increases activity of a NFκB promoter reporter; Chemotherapy-induced increase in PID1 mRNA is mitigated by NFκB inhibitors. (**A**) Anti-NFκB/DAPI immunocytochemistry of LN229 cells treated with etoposide (10 µg/ml) in serum-free medium for the indicated times. TNFα (150 ng/ml, 2 hrs) served as positive control. Upper panels shows fields with several representative cells. Lower panels show magnification of a representative cell from each of these fields. Scale bars are 20 µm. (**B**) Western blots (cropped) of cytosolic and nuclear fractions of LN229 GBM cells (2.5 × 10^6^ cells in 10 cm plate) treated with etoposide (20 µg/ml) for the indicated times. Nuclear fraction lanes were loaded with 5x more cell-equivalents compared to cytosolic fractions and required longer exposure to compensate for the lower nuclear NFκB amount. (**C**) LN229 GBM cells were transfected for 24 hrs with Renilla luciferase NFκB promoter reporter and Firefly luciferase control reporter and then incubated 24 hrs with cisplatin (5 µg/ml; (**C**), etoposide (10 µg/ml; (**E**) or vincristine (50 ng/ml; V). Reporter activity was measured after 24 hr incubation. Cisplatin *p* = 0.007 (n = 3), etoposide *p* = 0.002 (n = 3), vincristine *p* = 0.016 (n = 2). (**D**) LN229 transfected 24 hrs with Renilla pLightSwitch-PID1 promoter reporter or pLightSwitch control, along with pGL3P firefly luciferase were incubated for additional 24 hrs with etoposide (10 µg/ml) in presence of vehicle or JSH-23 (5 µM or 10 µM) in duplicate samples before measuring reporter activity. PID1 promoter reporter activity is depicted as fold change, normalized to 1 in controls. Control *vs*. etoposide p = 0.0026; etoposide *vs*. etoposide + JSH-23 (5 µM) p = 0.011, etoposide *vs*. etoposide + JSH-23 (10 µM) p = 0.013. (**E**,**F**) LN229 GBM (**E**) and UW228 medulloblastoma (**F**) cells were incubated 24 hrs with etoposide (10 µg/ml) or cisplatin (10 µg/ml) in presence of vehicle control or NFκB inhibitors JSH-23 (5 µM) or Bay117082 (1 µM). *PID1* and *GAPDH* mRNA were measured by qRT-PCR. (**G**) Activity of NFκB inhibitors was confirmed in 293 T cells transiently transfected (18 hrs) with the NFκB-SEAP reporter plasmid that were then treated with the NFκB inhibitors JSH-23 (5 µM or 10 µM) or Bay117082 (1 µM) or vehicle, and an hour later, with TNFα (20 ng/ml) or vehicle to activate NFκB. Twenty four hours after addition of TNFα or vehicle the medium was analyzed for secreted alkaline phosphatase activity. n = 3 experiments, bars are means ± SD.

Chemotherapy has been reported to increase and activate NFκB in a number of cancer cell lines^16^. The *PID1* promoter harbors putative recognition sites for NFκB, prompting us to examine possible involvement of NFκB in the *PID1* response to chemotherapy. Immunofluorescence staining showed that in resting LN229 GBM and UW228 medulloblastoma cells NFκB was mostly cytoplasmic, showing increase in nuclear localization upon etoposide exposure (Fig. 4A and Supplementary Fig. S4). Exposure to TNFα, a known inducer of NFκB translocation^17^, also caused nuclear translocation of NFκB and is shown for comparison (Fig. 4A). Western blotting of fractionated cell lysates similarly showed that LN229 GBM and UW228 medulloblastoma cells treated with etoposide increased nuclear NFκB compared to vehicle-treated cells (Fig. 4B and Supplementary Fig. S4). This was transient, and by 24 h the nuclear-translocated NFκB fraction was no longer apparent (Supplementary Fig. S4 and data not shown). Consistent with this, both etoposide and cisplatin increased NFκB luciferase reporter activity in LN229 cells (Fig. 4C) as well as in a cell line from a non-brain neural crest-derived cancer, childhood neuroblastoma (CHLA-255 cells, Supplementary Fig. S5), further supporting NFκB involvement in the response to chemotherapy.

Activity of a *PID1* promoter reporter was also increased by etoposide and was inhibitable by JSH-23 (5–10 µM), an inhibitor of nuclear translocation of NFκB (Fig. 4D). JSH-23 also diminished baseline and etoposide-induced increase in *PID1* mRNA in LN229 GBM cells (Fig. 4E) and suppressed the cisplatin-induced increase in *PID1* mRNA in UW228 medulloblastoma cells (Fig. 4F). A second NFκB inhibitor, Bay117082 (1 µM), which irreversibly inhibits NFκB translocation to the nucleus due to inhibition of phosphorylation of the NFκB inhibitory binding partner IκBα, also diminished the cisplatin-induced increase in *PID1* mRNA (Fig. 4F). Effect of these inhibitors on NFκB activity was validated using an NFκB reporter assay (Fig. 4G). Taken together, these data suggest that the etoposide- and cisplatin-induced increase in *PID1* mRNA in LN229 GBM and UW228 medulloblastoma cells is at least partly mediated by NFκB.

### PID1 protein is differentially regulated by etoposide and cisplatin

**Figure 5:**
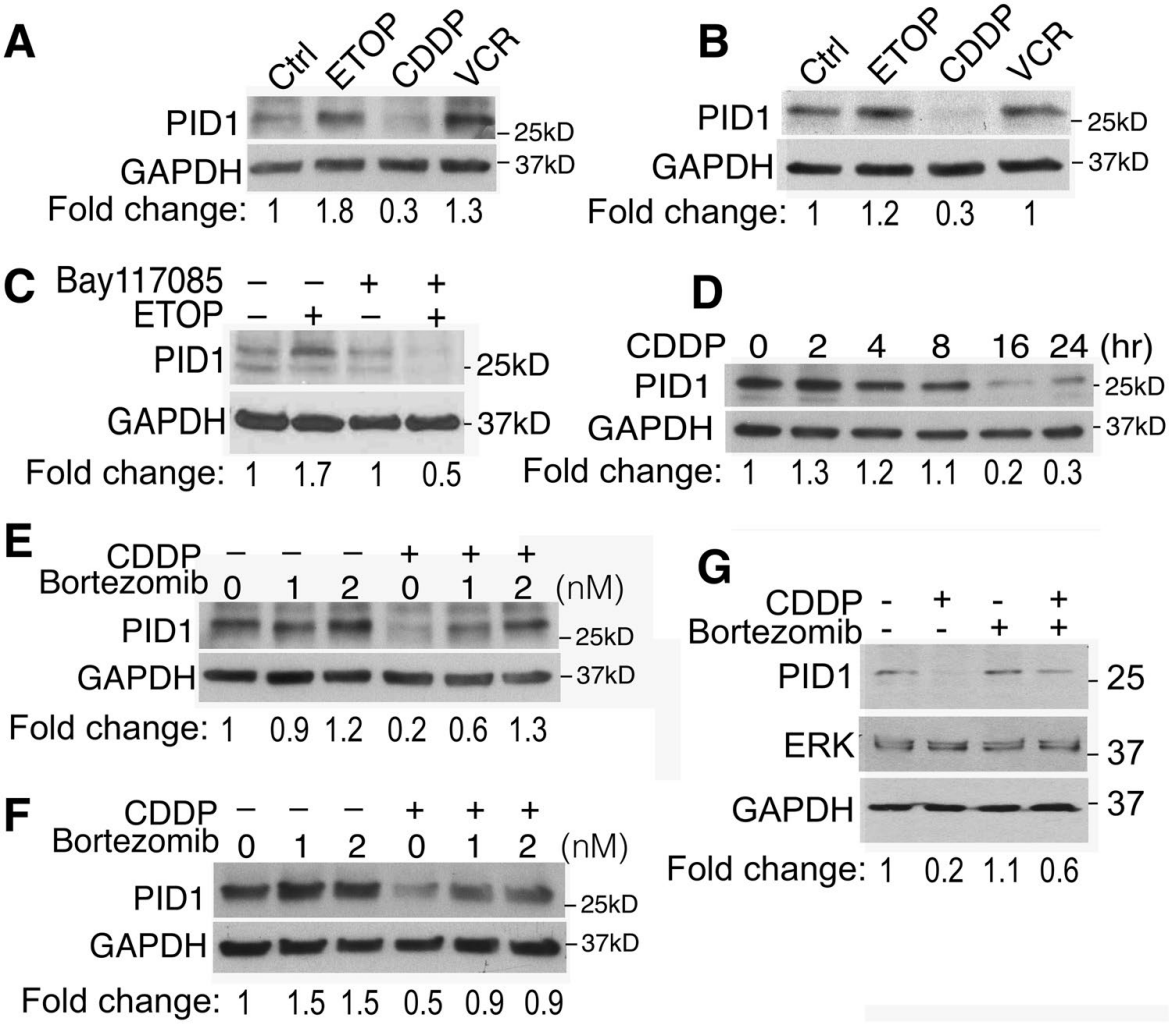
PID1 protein is differentially regulated by etoposide and cisplatin. SDS-PAGE and western blotting of tumor cell lysates for PID1 protein. (**A**,**B**) PID1 protein is increased by etoposide and vincristine but decreased by cisplatin: LN229 (**A**) or U87 (**B**) GBM cells were plated overnight in 6-well plates (4 × 10^5^ cells/well). Cells were then treated with vehicle, cisplatin (5 µg/ml; CDDP), etoposide (10 µg/ml; ETOP), or vincristine (50 ng/ml; VCR) for 24 hrs. (**C**) Etoposide-induced increase in PID1 protein is inhibited by the NFκB inhibitor, Bay117085: U87 cells were pre-treated with Bay117085 (2 µM) prior to exposure to etoposide (10 µg/ml) for 24 hr. Shown is one of two experiments with similar results. (**D**) Cisplatin decreases PID1 protein: U87 cells were treated with cisplatin (5 µg/ml) for the times indicated. (**E**,**F**) Bortezomib prevents the cisplatin-induced decrease in PID1 protein: Bortezomib was added to LN229 (**E**) and U87 (**F**) GBM cells 1 hr prior to start of a 24 hr incubation with cisplatin (5 µg/ml). (**G)** ERK and GAPDH protein levels are not decreased by cisplatin while PID1 is: Bortezomib (2 nM) or vehicle were added to U87 cells 1 hr before they began 24 hr incubation with cisplatin (5 µM). Densitometry normalized to controls in left lane is listed under the blot.

In Fig. 3 we showed that etoposide increased *PID1* mRNA. Western blotting shows a corresponding etoposide-induced increase in PID1 protein (Fig. 5A–C and data not shown; mean increase 1.62 fold ± 0.134, n = 4 experiments). Consistent with the effect of NFκB inhibitors on *PID1* promoter activity and *PID1* mRNA in Fig. 4D–F, induction of PID1 protein by etoposide was also inhibited by the NFκB inhibitor, Bay117085 (2 µM, U87 cells, Fig. 5C), supporting that NFκB mediates its induction.

Interestingly, despite the overall similar increase in *PID1* mRNA by etoposide and cisplatin (Fig. 3), whereas etoposide increased PID1 protein, cisplatin consistently decreased it, in both LN229 and U87 cells (Fig. 5A,B) as well as in UW228 (Supplementary Fig. S6). The decrease in PID1 protein began as early as 4 hrs after addition of cisplatin and was lowest by 16–24 hrs (Fig. 5D). The cisplatin-induced decrease in PID1 protein in the face of an increase in *PID1* mRNA suggested instability and/or degradation of PID1 protein following cisplatin. Consistent with this cisplatin can induce protein degradation via the ubiquitin-proteasome pathway^18–21^. We therefore tested the effect of bortezomib, a potent proteasome inhibitor approved for treatment of multiple myeloma and mantle cell lymphoma^22^ and tested in some brain tumor models^23, 24^. Bortezomib mitigated the cisplatin-induced decrease of PID1 protein in a dose-dependent manner in both glioma (LN229, U87) and medulloblastoma (UW228) cells (Fig. 5E–G, Supplementary Fig. S6A). The cisplatin-induced decrease in PID1 protein did not result from a general effect of cisplatin on all proteins, since ERK and GAPDH protein levels were unaffected by cisplatin and/or bortezomib (Fig. 5G). The cisplatin-induced decrease in PID1 protein level occurred even when PID1 was expressed in the pCIENS plasmid, where its expression is not driven by the endogenous promoter (Supplementary Fig. S6B), supporting that cisplatin has effect on stability of the PID1 protein itself. These data indicate that the cisplatin-induced decrease in PID1 protein may be mediated through proteasomal degradation.

Taken together, our results demonstrate that (1) PID1 sensitizes glioma and medulloblastoma cell lines to cisplatin and etoposide and mediates at least part of their cisplatin-induced apoptosis and mitochondrial depolarization; (2) etoposide, cisplatin and vincristine increase *PID1* mRNA in glioma and medulloblastoma cell lines; etoposide and cisplatin increase NFκB promoter reporter activity and cause nuclear translocation of NFκB, and *PID1* mRNA is increased in a NFκB-dependent manner; (3) etoposide increases PID1 protein consistent with its effect on mRNA, but the cisplatin-induced increase in *PID1* mRNA is accompanied by decrease in PID1 protein, whose level can be restored by bortezomib. Thus, PID1 sensitizes glioma and medulloblastoma cell lines to etoposide and cisplatin in a mechanism that involves NFκB.

## Discussion

**Figure 6:**
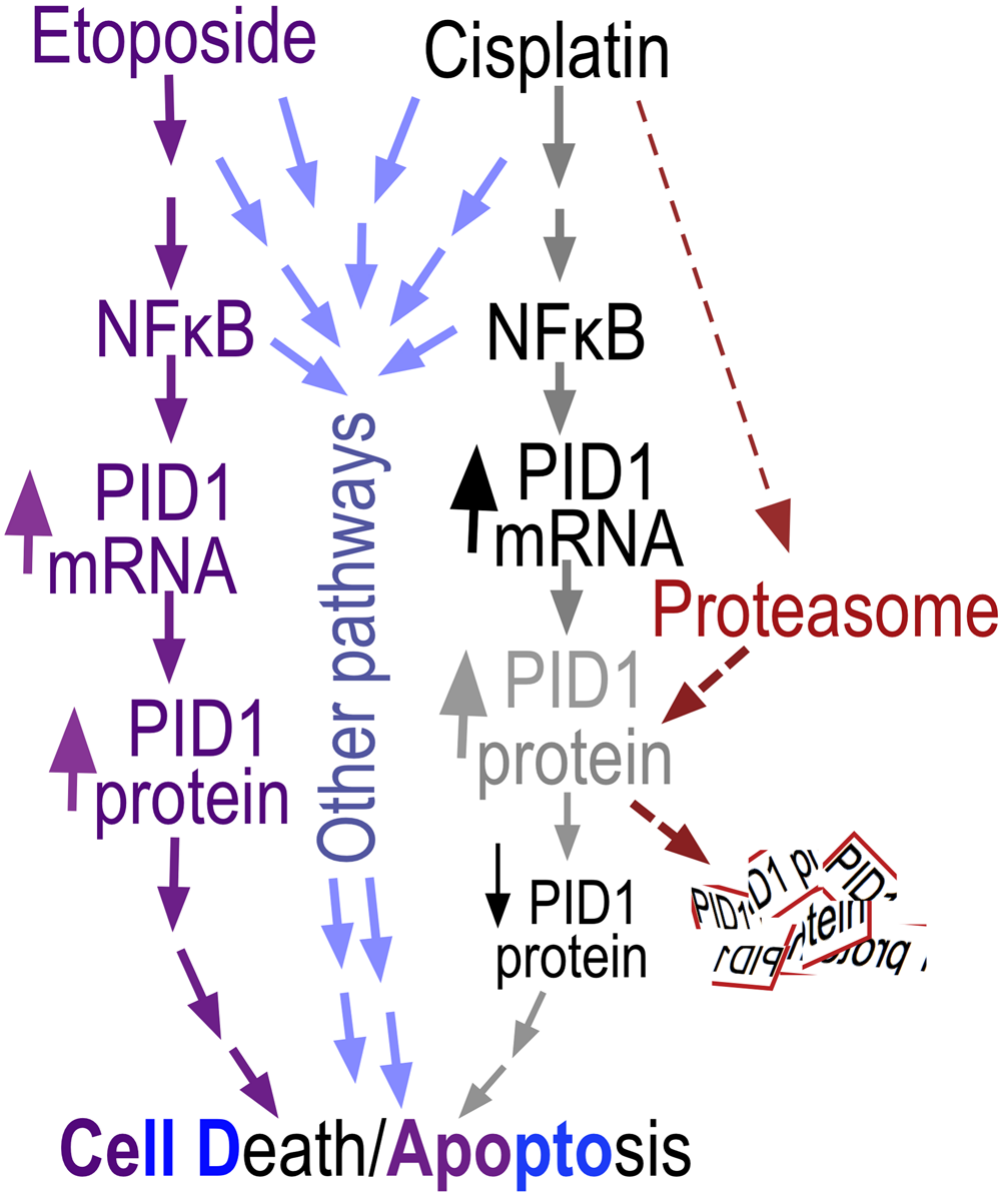
PID1 in chemotherapy-induced medulloblastoma and glioma apoptosis. The data suggest that PID1 contributes to chemotherapy-induced apoptosis through an NFκB-dependent mechanism as follows: Etoposide and cisplatin both increase *PID1* mRNA via NFκB and both increase apoptosis. While etoposide increases both *PID1* mRNA and PID1 protein, cisplatin increased *PID1* mRNA, but decreased PID1 protein. The cisplatin-induced decrease in PID1 protein is reversed by bortezomib, suggesting that it is due to proteasomal degradation. It is likely that both etoposide and cisplatin activate additional signaling pathways that contribute to apoptosis.

We tested the hypothesis that PID1 enhances chemotherapy-induced cell death in brain tumor cell lines. Our findings in medulloblastoma and glioma cells show for the first time that PID1 increases apoptosis induced by etoposide and cisplatin, that siRNA-mediated downregulation of PID1 suppresses cisplatin-mediated apoptosis, and that etoposide and cisplatin increase *PID1* mRNA in an NFκB-dependent manner, suggesting that PID1 mediates part of the responsiveness of these brain tumor cell lines to chemotherapy (Fig. 6). These results are consistent with the correlation between higher level of tumor *PID1* mRNA and longer survival of medulloblastoma and glioma patients (ref. 7 and Supplementary Fig. S1). In this respect, it will be interesting to examine if PID1 augments and/or mediates response of medulloblastoma and glioma cells to irradiation and/or temozolomide, which are used in therapy of these tumors.

We observed increased activity of the NFκB promoter following etoposide and cisplatin and translocation of NFκB to the nucleus following etoposide. This is consistent with reports, including in brain tumor cells, that chemotherapy can induce NFκB promoter activity, NFκB translocation to the nucleus, and other aspects of NFκB activation^16, 25^. NFκB activation can have opposite effects: while it can mediate cell kill in response to chemotherapy, it can also mediate tumor growth and contribute to development of resistance to chemotherapy^16, 26, 27^. In our cultured medulloblastoma (UW228), glioma (LN229) and neuroblastoma (CHLA-255) cell lines the etoposide- and cisplatin-induced increase in PID1 mRNA was blocked by NFκB inhibitors, suggesting that it was mediated by NFκB.

Both cisplatin and etoposide increased *PID1* mRNA, but only etoposide increased PID1 protein, while cisplatin decreased it (Figs 3, 4 and 5). The mitigation of cisplatin-induced decrease in PID1 protein by bortezomib, a proteasome inhibitor, (Fig. 5E–G) suggests that cisplatin induces proteasome-mediated degradation of PID1 protein. The requirement for PID1 in cisplatin-induced apoptosis indicated by the siRNA experiments (Fig. 2) suggests this proteasome-mediated decrease in PID1 may be a compensatory cell survival mechanism upon exposure to cisplatin. This is supported by reports that cisplatin can activate ubiquitination and proteasomal degradation of several proteins involved in chemotherapy-induced apoptosis in a mechanism thought to induce resistance to the anti-tumor effects of cisplatin^19, 21^. Proteasome inhibitors augment the apoptotic response to cisplatin in several cancers, including GBM cell lines, and are suggested as a way to augment efficacy of therapy^26, 28^. It will be interesting to examine if emergence of cisplatin resistance is linked to this decrease in PID1 protein and if blocking the degradation will prevent or reverse such resistance^18, 26, 28–30^.

Taken together, our results demonstrate for the first time that PID1 increases apoptotic response of glioblastoma and medulloblastoma cell lines to cisplatin and etoposide and that PID1 is required for cisplatin-mediated apoptosis. This suggests that the direct correlation between higher *PID1* mRNA levels and longer patient survival may reflect a PID1-mediated relative sensitivity of these cancers to therapy. This finding suggests that a more thorough understanding of PID1 biology will forward development of new approaches to brain tumor therapy.

## Materials and Methods

### AnnexinV, mitochondrial depolarization and caspase 3 cleavage

Analyses were performed on the green fluorescent protein (GFP)-positive cells 48 hrs after transfections with pCMV-PID1-IRES-eGFP﻿ or pCMV-IRES-eGFP control﻿ and 24 hrs after drug treatment. AnnexinV/7AAD staining was by flow cytometry using the APC AnnexinV kit (BD Pharmingen catalog #550474) according to manufacturer’s instructions. Mitochondrial depolarization was measured by flow cytometry using the MitoProbe™ DiIC1(5) Assay Kit for Flow Cytometry (Life Technology catalog #M34151). Caspase 3 cleavage staining was assessed using anti-cleaved-caspase 3-pacific blue antibody (Cell Signaling catalog #8788) by flow cytometry. Flow cytometry analysis was performed using an SLR II flow cytometer (BD Biosciences). Cell clumps and sub-cellular debris were excluded using appropriate gating on forward and side light scatter. Data were analyzed using FACSDIVA software (BD Biosciences).

### Cell Culture

Cell lines used were D283MED Group 3 medulloblastoma and UW228-2 SHH medulloblastoma (called here UW228)^31^, and LN229, U87, LN18 GBM cells (ATCC). D54 GBM cells were a kind gift from Dr. Darell Bigner (Duke University, Durham, NC). Cell lines were used less than 2–3 months after thawing and/or less than 50 passages from the original vial obtained and were authenticated. LN229, LN18 and U87 were maintained in DMEM with 10% fetal bovine serum (FBS); D54 were maintained in 10% FBS DMEM with 0.2% sodium bicarbonate (15 ml of 7.5% solution per 500 ml, GIBCO 2508) per 500 ml medium; UW228 and D283MED were maintained in 1:1 DMEM/F-12 with 10% FBS. For transfections, cells were seeded in 6-well plates at 4 × 10^5^ cells/well the day before transfection. Cells approaching 80–90% confluence were transfected with 1 μg plasmid and 5 μl Lipofectamine2000 (Life Technology) in 1 ml serum-free medium in 6-well plates for 4–6 hrs according to manufacturer’s instructions and then grown in regular growth medium for additional 18 hours.

### Cell Imaging

Cells were fixed in 3.7% formaldehyde for 20 min and incubated for 1 hr in a 1:300 dilution of anti-NFκB p65 rabbit monoclonal IgG (Cell Signalling catalog #4764) followed by 1 hr in 1:500 FITC-conjugated anti-rabbit IgG (Jackson ImmunoResearch Laboratories) with 60 nM 4′,6-diamidino-2-phenylindole (DAPI; Life Technologies/Thermo Fisher Scientific). Images were acquired with a DMI6000B microscope equipped with an HC PLAN APO 20x/0.7 Ph2 objective lens (Leica Microsystems) and ORCA-Flash4.0 LT camera (Hamamatsu Photonics).

### Luciferase promoter reporter assays

Tumor cells plated in 6-well plates overnight were co-transfected with either empty vector control pLightSwitch-Prom (0.2 µg, SwitchGear Genomics, catalog #S790005) or human PID1 luciferase promoter-reporter pLightSwitch-PID1 (0.2 µg, SwitchGear Genomics, catalog #S708635; optimized Renilla) together with pGL3P firefly luciferase control vector (4 ng) using Lipofectamine 2000 (Life Technology, catalog #11668027). Starting 24 hrs after transfection cells were treated for 24 hrs as indicated and were collected 48 hrs after transfection for assessment of promoter-reporter luciferase activity. Luciferase assay was performed using Dual-Luciferase® Reporter Assay (Promega, catalog #E1910) following manufacture’s instruction. Relative Renilla luciferase activity in the extracted protein samples was measured in triplicates and normalized against Firefly luciferase activity from the co-transfected pGL3P control vector.

### NFκB activity assay

293 T cells were transfected (0.125 µg/6-well) with pNFκB-SEAP plasmid (Clontech cat #631905) for 6 hrs. Cells were then placed in medium with heat inactivated FBS (to inactivate alkaline phosphatase in the medium). NFκB inhibitors or vehicle were added to the cells and an hour later, TNFα or vehicle for another 24 hrs. Secreted alkaline phosphatase activity was measured in aliquots of the conditioned medium using the NBP2-25286 NFkB Secreted Alkaline Phosphatase Assay Kit (NovusBio).

### Plasmids

*PID1* (NCBI NM_001100818.1, variant 2) cloned into EcoRI and XhoI in pCIENS^7^ (a CMV promoter-driven expression vector that also expresses eGFP via an EMCV IRES) was used to express PID1 in all experiments (pCIENS-PID1-IRES-eGFP). pCIENS-IRES-eGFP without *PID1* was used for control transfections.

### Quantitative RT-PCR

Quantitative real-time PCR was performed using the Applied Biosystems 7900HT sequence detection system (Applied Biosystems) as described^7^. PCR primers and probes were designed and synthesized using Primer Express software (Applied Biosystems) or SciTools (Integrated DNA Technologies). Primers and probes, *PID1*: forward primer: 5′-AGCCAGTCATTGAGCTCTGGAAGA-3′, reverse primer: 5′-TGGTCGAGATGATGGAGCCAAACT-3′, probe: 5′-TTTCCGGCCAATGCCCTCCTGGAAAT-3′; GAPDH: forward primer: 5′-CAACTACAT GGTTTACATGTTCCAATATG-3′, reverse primer: 5′-GGGATCTCGCTCCTGGAAG-3′, probe: 5′-CGTTCTCAGCCTTGACGGTGCCA-3′. TaqMan real-time PCR data were analyzed using ABI Sequence Detector Software. *PID1* mRNA was normalized to GAPDH mRNA, which was quantified in parallel in each sample.

### Reagents

The proteosomal inhibitor Bortezomib (catalog #T4376-100MG) and NFκB inhibitors Bay117082 (catalog #B5556-10MG) and JSH-23 (catalog #J4455-5MG) were from Sigma-Aldrich Co. Other reagents are also from Sigma-Aldrich Co unless otherwise specified.

### siRNA transfections

A mixture of two *PID1*-specific siRNAs (GGGAGATCAATGATGACCTGT and GGAATTGGAATCCGATGATGG) designed using the siRNA Selection Program at http://sirna.wi.mit.edu/reference.php was used as described^7^ at a 1:1 ratio. Non-silencing negative control siRNA was from Qiagen. FAM-labeled non-silencing siRNA negative control was from Life Technologies. For transfection, si*Control* (mix of 100 nM non-silencing siRNA + 10 nM FAM-labeled non-silencing siRNA control) or si*PID1* (100 nM of 1:1 mix of the two *PID1*-specific siRNAs + 10 nM FAM-labeled non-silencing siRNA control) were transfected using lipofectamine2000 for 5 hrs. Validation of *PID1* knockdown was by western blot and qRT-PCR as in ref. 7.

### Western Blotting

Whole cell lysates were resolved by SDS-PAGE (4–12% gradient gel, BioRad) and proteins were identified by western blotting as described^32^. Anti-PID1 rabbit polyclonal antibody (Sigma-Aldrich catalog #HPA036103) was used at 1:1,000 as described^7^. Anti-GAPDH mouse monoclonal (1:20,000) and anti-ERK rabbit antibody (1:1000) were from Santa Cruz Biotechnology, CA. Blots were scanned into pdf files, opened in Photoshop, and exposure level adjusted equally across the whole blot (usually lighter by 0.5–0.75). Uncropped western blots are provided in Supplementary Data. Densitometry was performed using ImageJ software^33^.

### Statistical analysis

*In vitro* experiments were analyzed using GraphPad Prism version 6.0b for Mac (GraphPad Software; www.graphpad.com). Results are depicted as mean ± SEM from at least 3 independent experiments unless stated otherwise. P-values represent unpaired 2-sided Student t-test unless stated differently. Kaplan Meier curves of the glioblastoma datasets from The Cancer Genome Atlas cohort of patients (n = 540, of which 37 cases were not included due to lack of clinical data) and the Tumor Glioma – French – 284 Mas5.0 – u133p2 dataset (n = 284 with 11 cases not included due to lack of clinical data) were analyzed through the R2 Genomic Analysis Visualization Platform (http://r2.amc.nl) and their p-values were calculated by the R2 platform user interface.

## Electronic supplementary material

Supplementary Figures
