# Supplementary material for: PID1 increases chemotherapy-induced apoptosis in medulloblastoma and glioblastoma cells in a manner that involves NFκB
Source: Sci Rep. 2017 Apr 11;7:835. doi: 10.1038/s41598-017-00947-6 (PMC5429784; doi:10.1038/s41598-017-00947-6)

SUPPLEMENTARY FIGURES:

PID1 increases chemotherapy-induced apoptosis in medulloblastoma and glioblastoma cells in a manner that involves NFκB

Jingying Xu, Xiuhai Ren, Anup Singh Pathania, G. Esteban Fernandez, Anthony Tran, Yifu Zhang, Rex A. Moats, Gregory M. Shackleford and Anat Erdreich-Epstein

Supplementary Figure S1

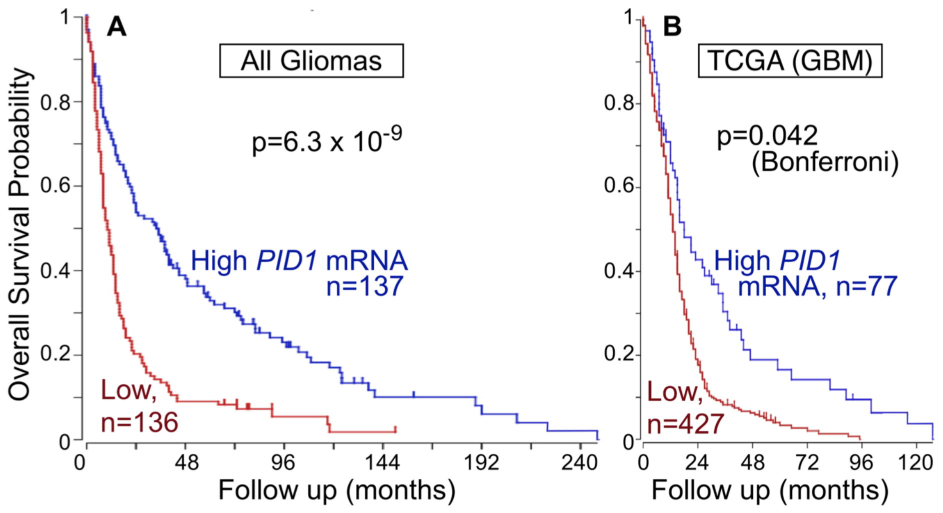

Supplementary Figure S1: Higher *PID1* mRNA correlates with longer overall survival in gliomas and glioblastomas. Kaplan Meier curves of overall survival (OS) according to *PID1* mRNA in tumors at diagnosis (219093_at) through the R2 Microarray Portal (http://hgserver1.amc.nl/cgi-bin/r2/main.cgi). **A)** 273 patients with any glioma from the dataset Tumor Glioma - French - 284 MAS5.0-u133p2 accessed February 26, 2015 (11 patients of the cohort of 284 were censored due to not having survival data)[35]. *p*-value = 6.3×10^{-9} between tumors with higher than median tumor *PID1* mRNA level (n=137, blue curve) and lower-than-median (n=136, red curve); **B)** 504 patients with GBM from dataset Tumor Glioblastoma - TCGA - 540 - Mas5.0 - u133a accessed December 30, 2014 (36 of the cohort of 540 were censored due to not having survival data). p =0.042, after Bonferroni correction for multiple analyses (raw p-value: 8.5×10^{-5} before correction); n=77 in the 'high' *PID1* group (blue), n=427 in the 'low' group (red).

Supplementary Figure S2

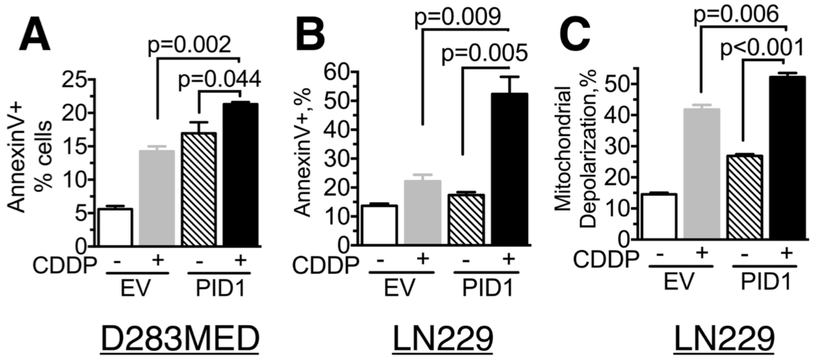

Supplementary Figure S2: Overexpressed PID1 increases apoptosis in D283 medulloblastoma and LN229 glioma cells treated with cisplatin. A) D283 medulloblastoma cells treated with cisplatin (10 μM) as in Fig 1A and analyzed for apoptosis by AnnexinV/7AAD; values shown are for the upper right and lower right quadrants, indicative of early+late apoptosis. Shown are means±SEM from one of three experiments performed in 2-3 replicates with similar results. **B-C)** LN229 GBM cells treated with cisplatin (10 μM) as in Fig 1 and analyzed for apoptosis by AnnexinV/7AAD (**B**) and mitochondrial depolarization (**C**). Shown are means±SEM from one of three (B) or two (C) experiments performed in 2-3 replicates with similar results.

Supplementary Figure 3

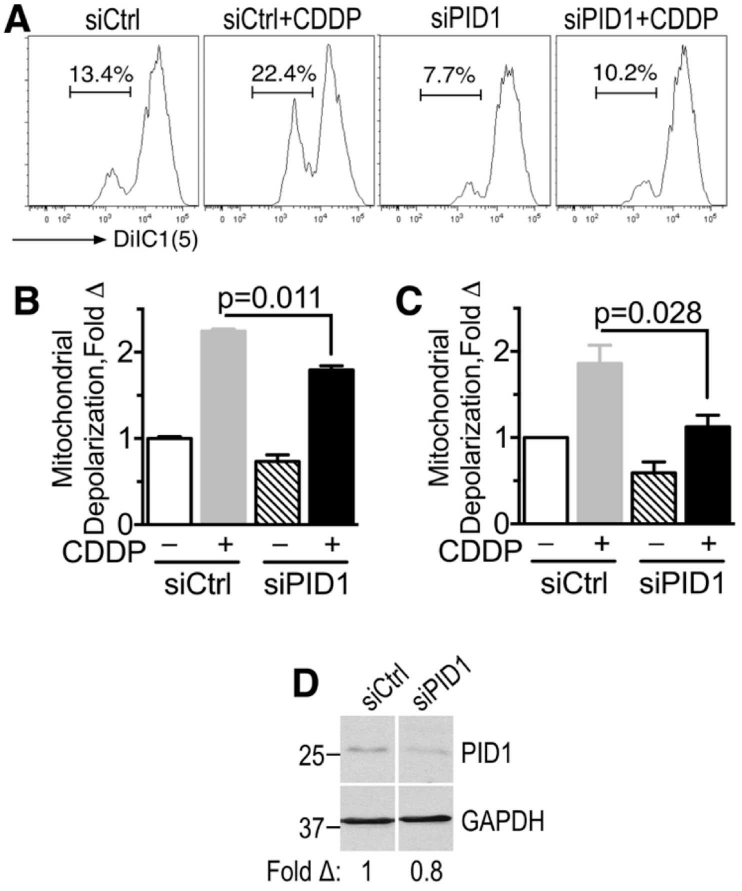

Supplementary Figure S3: siRNA to PID1 mitigates cisplatin-induced mitochondrial depolarization in D283 medulloblastoma and U87 glioblastoma cells. Cells were transfected with non-silencing control siRNA (siCtrl) or PID1 siRNA (siPID1), along with FAM-labeled non-silencing siRNA to monitor transfection efficiency. Twenty-four hrs after transfection cells were treated with 10 µg/ml cisplatin for 24 hrs and mitochondrial membrane depolarization (DiIC1(5)) was assessed by flow cytometry in the FAM-labeled cells. **A-B)** D283 medulloblastoma; **A)** representative curve, **B)** Mean±SEM (n=3 replicates) from one of three experiments with similar results. **C)** U87 GBM cells, mean±SEM of four experiments performed in 1-3 replicates. **D)** Representative western blot of PID1 in UW228 cells after siRNA knockdown comprising of lysates from the transfected wells without selection or sorting. Flow cytometry of the FAM labeled cells indicates that 60-70% of these cells took up the FAM label (and the siRNA), suggesting that PID1 knockdown per FAM-labeled cell is approximately 50%.

Supplementary Figure S4

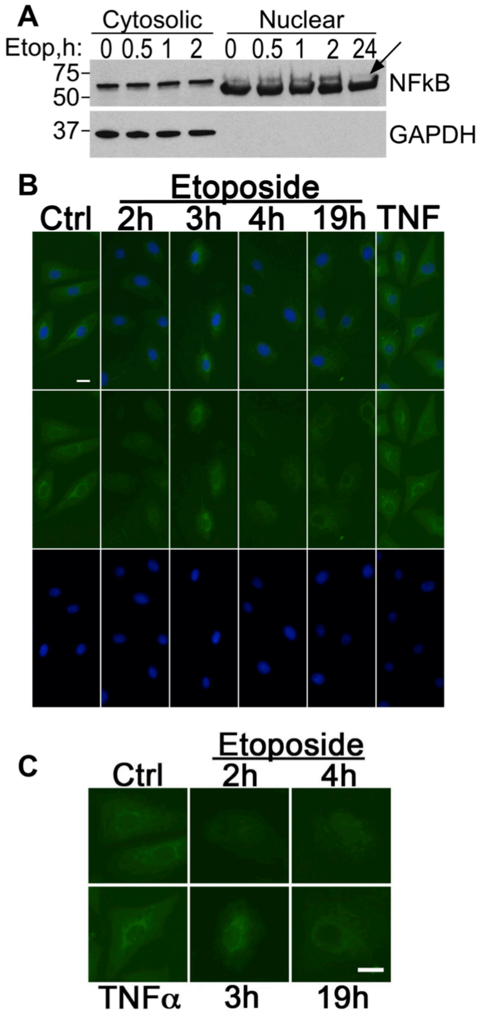

Supplementary Figure S4: Etoposide induces nuclear translocation of NFκB in UW228 medulloblastoma cells. UW228 cells were treated for the indicated times as in Figs 4A-B and analyzed for nuclear translocation of NFκB. (**A**) Western blotting (ratio of loaded cytosolic and nuclear cell-equivalents was 1:22; translocated NFκB is the upper band (shown by the arrow), which initially increases, but by 24 hrs disappears. (**B**) Immunofluorescence microscopy as in Fig 4. TNFα positive control was 150 ng/ml for 19 hrs. Scale bar = 20 μm.

Supplementary Figure S5

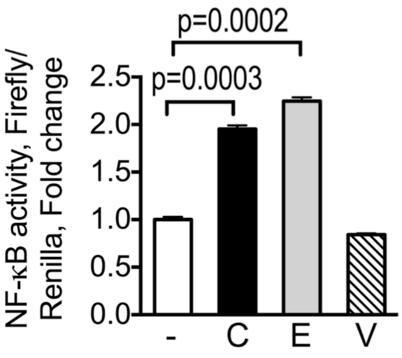

Supplemental Figure S5: Chemotherapy induces NFκB promoter activity in CHLA-255 neuroblastoma cells. CHLA255 neuroblastoma cells were transfected with Renilla luciferase NFκB promoter reporter firefly control reporter for 24 hrs and then treated with cisplatin (5 μg/ml; C), etoposide (10 μg/ml; E), and vincristine (50 ng/ml; V) for 24 hrs, followed by measurement of promoter reporter activity as described in Fig 4. n=3.

Supplementary Figure S6

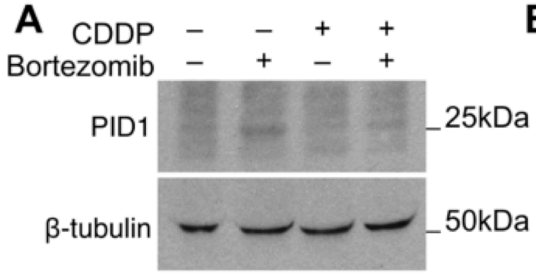

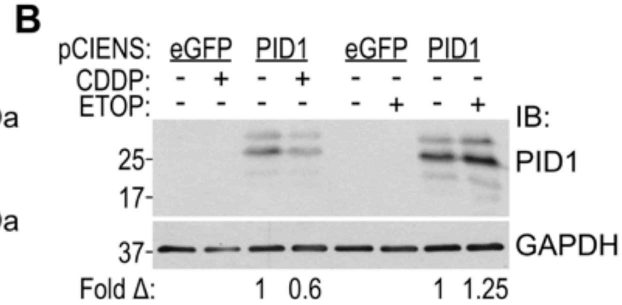

Supplementary Figure S6: Cisplatin decreases PID1 protein in UW228 medulloblastoma cells and bortezomib partially restores its level. A) UW228 medulloblastoma cells were pre-incubated for 1 hr with 2 nM bortezomib prior to addition of 5 μM cisplatin for 24 hrs. Lysates were analyzed by western blotting. **B)** UW228 cells were incubated with 10 μM cisplatin (CDDP) or 5 μM etoposide (ETOP) for 24 hrs. Lysates were analyzed by western blot and densitometry calculated relative to GAPDH to provide fold-change in PID1 protein in presence of chemotherapy vs. vehicle control.

Supplementary Figure S7

Full blots, for Fig 4B

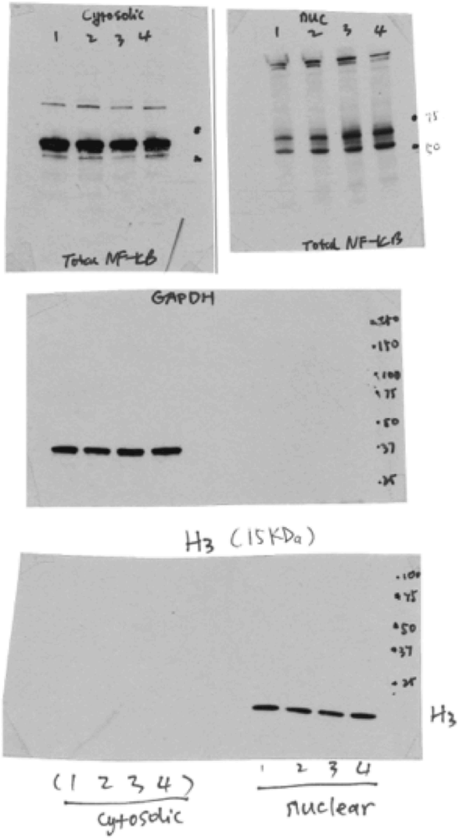

Supplementary Figure S8

Full blots, for Fig 5B

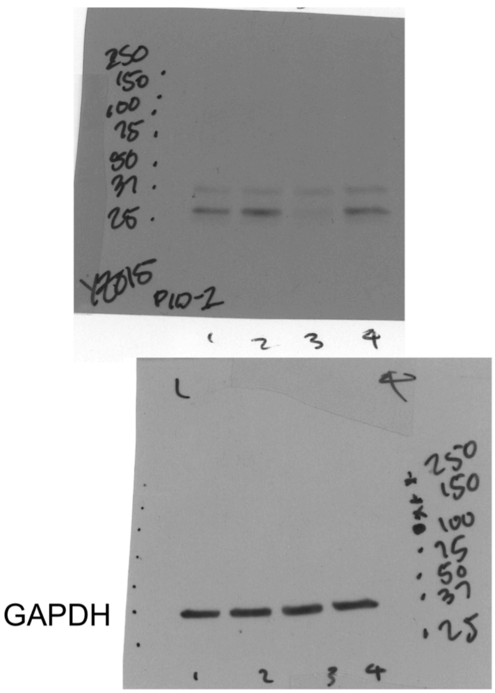

Supplementary Figure S9

Full blot for Fig 5A

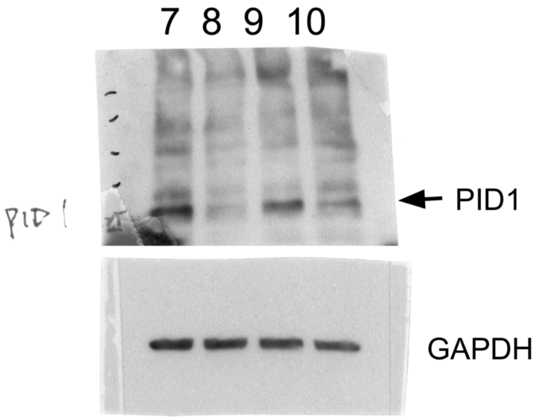

(Flipped horizontal
compared to panel 5A)

Supplementary Figure S10

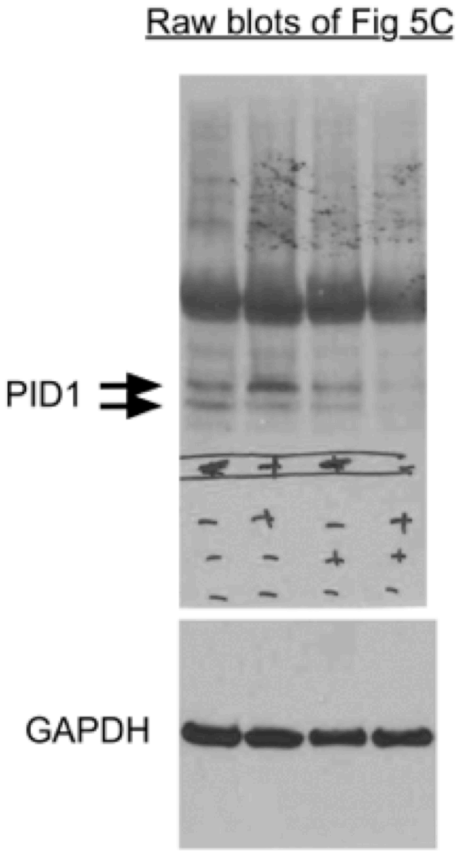

Supplementary Figure S11

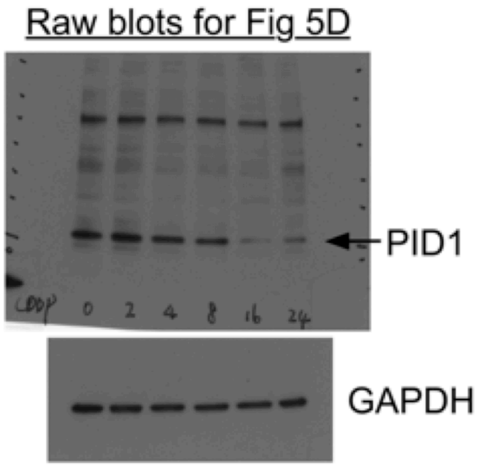

Supplementary Figure S12

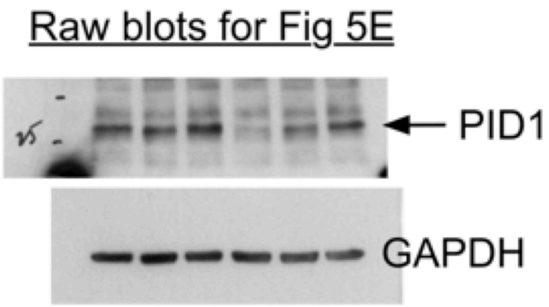

Supplementary Figure S13

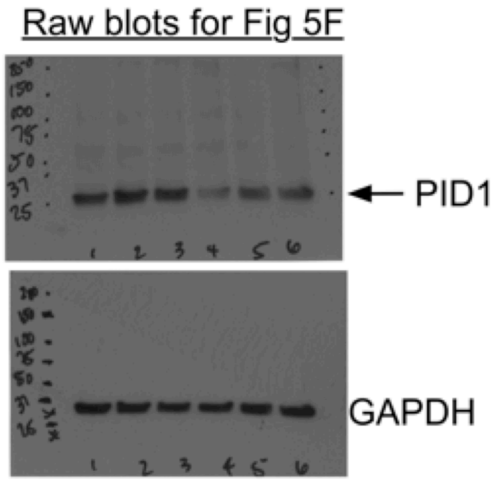

Supplementary Figure S14

Raw blots for Fig 5G

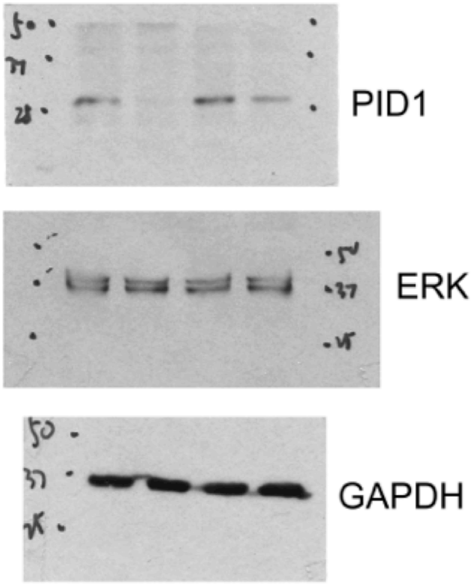

Supplement: Supplementary file 1 — Supplementary Figures [file 41598_2017_947_MOESM1_ESM.pdf]
